# Supplementary figures and images for: H2O2 repurposes plant O2 sensing to regulate post-hypoxia responses
Source: Nature. 2026 Apr 22;653(8116):1130–8. doi: 10.1038/s41586-026-10366-1 (PMC13216066; doi:10.1038/s41586-026-10366-1)

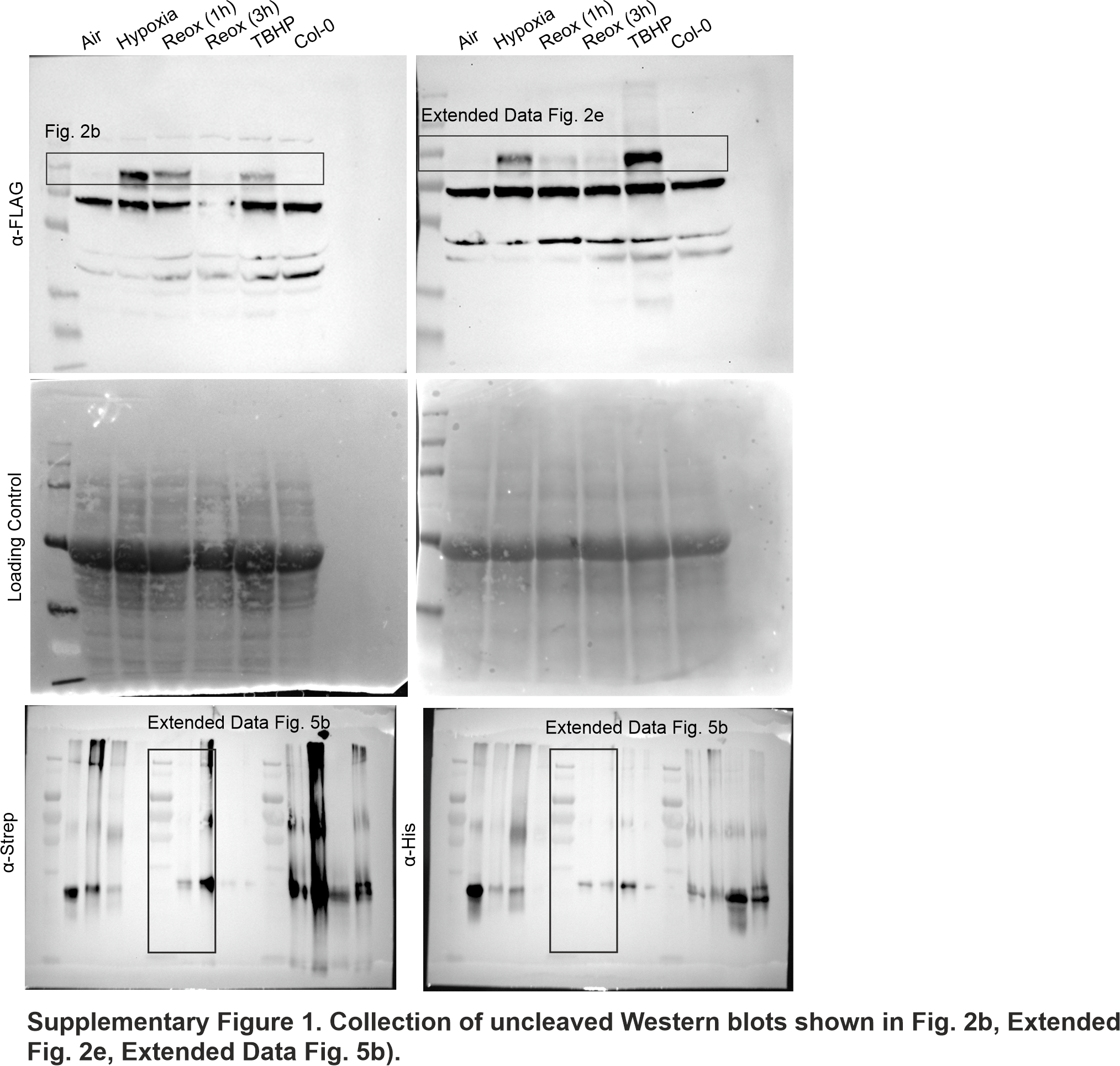

Supplement: Supplementary file 1 — Collection of uncropped western blot photos shown in Fig. 2b and Extended Data Figs. 2e and Fig. 5b. [file 41586_2026_10366_MOESM1_ESM.tif]
