# Supplementary material for: H2O2 repurposes plant O2 sensing to regulate post-hypoxia responses
Source: Nature. 2026 Apr 22;653(8116):1130–8. doi: 10.1038/s41586-026-10366-1 (PMC13216066; doi:10.1038/s41586-026-10366-1)
Supplement: Supplementary file 2 — Reporting Summary [file 41586_2026_10366_MOESM2_ESM.pdf]

nature portfolio

Corresponding author(s): Emily Flashman
Francesco Licausi

Last updated by author(s): Jan 7, 2026

Reporting Summary

Nature Portfolio wishes to improve the reproducibility of the work that we publish. This form provides structure for consistency and transparency in reporting. For further information on Nature Portfolio policies, see our Editorial Policies and the Editorial Policy Checklist.

Statistics

For all statistical analyses, confirm that the following items are present in the figure legend, table legend, main text, or Methods section.

n/a	Confirmed	
☐	☒	The exact sample size (*n*) for each experimental group/condition, given as a discrete number and unit of measurement
☐	☒	A statement on whether measurements were taken from distinct samples or whether the same sample was measured repeatedly
☐	☒	The statistical test(s) used AND whether they are one- or two-sided<br>*Only common tests should be described solely by name; describe more complex techniques in the Methods section.*
☒	☐	A description of all covariates tested
☒	☐	A description of any assumptions or corrections, such as tests of normality and adjustment for multiple comparisons
☐	☒	A full description of the statistical parameters including central tendency (e.g. means) or other basic estimates (e.g. regression coefficient) AND variation (e.g. standard deviation) or associated estimates of uncertainty (e.g. confidence intervals)
☐	☒	For null hypothesis testing, the test statistic (e.g. *F*, *t*, *r*) with confidence intervals, effect sizes, degrees of freedom and *P* value noted<br>*Give P values as exact values whenever suitable.*
☒	☐	For Bayesian analysis, information on the choice of priors and Markov chain Monte Carlo settings
☒	☐	For hierarchical and complex designs, identification of the appropriate level for tests and full reporting of outcomes
☐	☒	Estimates of effect sizes (e.g. Cohen's *d*, Pearson's *r*), indicating how they were calculated

Our web collection on statistics for biologists contains articles on many of the points above.

Software and code

Policy information about availability of computer code

Data collection	LC-MS/MS analysis of H2O2-treated AtPCO4: mass spectrometry data were acquired using the Orbitrap Eclipse mass spectrometer. Instrument control was through Orbitrap Eclipse Tune 3.5/3.1 and Xcalibur 4.5/4.4. RapidFire MS analysis of RAP22-15 oxidation: mass spectrometry data were acquired using an Agilent RapidFire RF360 sampling robot connected to an Agilent 6530 Accurate-Mass Q-ToF mass spectrometer. Spectra were visualised on Qualitative Analysis (version B.07.00).<br>Colorimetric intensities were collected with a EPSON Perfection V750 PRO scanner.<br>Histochemical staining was conducted with a Leica M165C stereo microscope<br>Confocal imaging was conducted with ZEISS LSM 880 Airyscan microscope (ZEN Lite software (version 3.11)).<br>Ratiometric readout of H2O2 biosensor was performed using a multiwell fluorimeter ClarioStar Plus (BMG Labtech).<br>RNA sequencing: Illumina Sequencing PE150 program on the NovaSeq 6000 platform (Novagene).
Data analysis	Data were analysed using GraphPad Prism 10.2.3(403) and R Statistical Software (version 4.3.1, Foundation for Statistical Computing, Vienna, Austria).<br>Image analysis with ImageJ (version 1.54j).<br>Confocal images were analysed using ZEISS ZEN Lite software (version 3.11)<br>RapidFire MS data were analysed using Agilent RapidFire Integrator (version 4.3.0.17235) to calculate integrated peak areas.<br>Peptide fragmentation by LC-MS/MS: peptide analysis was conducted using Peaks v. 8.5 and peptides were compared to predicted fragment patterns calculated using the University of California, San Francisco webpage tool Protein Prospector version 6.3.1.<br>Transcriptomic analyses (conducted in R software version 4.3.1) were aligned on the Arabidopsis thaliana full genome using Rsubread (version

2.16.1) and counted using featureCounts software (within the Rsubread package). Differentially expressed genes were identified using edgeR version 3.42.4. GO term enrichment analysis was conducted using clusterProfiler version 4.10.1.
DNA motif discovery was conducted on STREME (Sensitive, Thorough, Rapid, Enriched Motif Elicitation) and compared to known motif databases using Tomtom within the MEME Suite (version 5.5.9).

For manuscripts utilizing custom algorithms or software that are central to the research but not yet described in published literature, software must be made available to editors and reviewers. We strongly encourage code deposition in a community repository (e.g. GitHub). See the Nature Portfolio guidelines for submitting code & software for further information.

Data

Policy information about availability of data

All manuscripts must include a data availability statement. This statement should provide the following information, where applicable:

- Accession codes, unique identifiers, or web links for publicly available datasets
- A description of any restrictions on data availability
- For clinical datasets or third party data, please ensure that the statement adheres to our policy

RNA sequencing raw data generated for this study has been deposited in the Sequence Read Archive (SRA) at the National Centre for Biotechnology Information under BioProject ID PRJNA1380489 and PRJNA1171625 for RNA-sequencing of reoxygenation and oxidative stress, respectively. Numerical data used to generate the graphs displayed in Figures and Extended Data Figures are provided as Source Data supplementary files. Full version of all images are available at https://doi.org/10.5281/zenodo.18723507.

Research involving human participants, their data, or biological material

Policy information about studies with human participants or human data. See also policy information about sex, gender (identity/presentation), and sexual orientation and race, ethnicity and racism.

Reporting on sex and gender	N/A
Reporting on race, ethnicity, or other socially relevant groupings	N/A
Population characteristics	*Describe the covariate-relevant population characteristics of the human research participants (e.g. age, genotypic information, past and current diagnosis and treatment categories). If you filled out the behavioural & social sciences study design questions and have nothing to add here, write "See above."*
Recruitment	N/A
Ethics oversight	N/A

Note that full information on the approval of the study protocol must also be provided in the manuscript.

Field-specific reporting

Please select the one below that is the best fit for your research. If you are not sure, read the appropriate sections before making your selection.

☒ Life sciences ☐ Behavioural & social sciences ☐ Ecological, evolutionary & environmental sciences

For a reference copy of the document with all sections, see nature.com/documents/nr-reporting-summary-flat.pdf

Life sciences study design

All studies must disclose on these points even when the disclosure is negative.

Sample size	Described in each figure legend. Sample size was defined based on the power of statistical analysis to be used afterwards and on the limitations imposed by sample handling.
Data exclusions	No data were excluded
Replication	RNA experiments and BioDiaAlk probes were performed once. All other experiments were repeated twice, with similar results confirming replicability
Randomization	Plants were randomly assorted at growth facilities and their position randomly permutated to account for covariates (light and temperature gradients). (Bio)chemical and molecular analyses did not require randomisation to account for covariates due to the homogenous conditions<br>on lab benches.
Blinding	Investigators were not blinded but this should have no or minimal effect to the outcome of the experiments described in this manuscript.

Reporting for specific materials, systems and methods

We require information from authors about some types of materials, experimental systems and methods used in many studies. Here, indicate whether each material, system or method listed is relevant to your study. If you are not sure if a list item applies to your research, read the appropriate section before selecting a response.

Materials & experimental systems

n/a	Involved in the study	
☐	☒	Antibodies
☐	☒	Eukaryotic cell lines
☒	☐	Palaeontology and archaeology
☒	☐	Animals and other organisms
☒	☐	Clinical data
☒	☐	Dual use research of concern
☐	☒	Plants

Methods

n/a	Involved in the study	
☒	☐	ChIP-seq
☒	☐	Flow cytometry
☒	☐	MRI-based neuroimaging

Antibodies

Antibodies used	ANTI-FLAG® M2-Peroxidase (HRP) antibody (Sigma-Aldrich, cat. No.A8592) 1:5000<br>anti-streptavidin-HRP RABHRP3(Sigma-Aldrich) 1:10000<br>anti-GFP (Roche) cat. No. 11814460001 5.5 ng/uL<br>anti-his-HRP HRP-66005 (Proteintech) 1:1000
Validation	ANTI-FLAG antibodies were validated including negative controls that did not express the protein of interest (such negative controls are included in Fig. 2b and Extended Data Fig. 2e.). Anti-GFP antibodies were validated in doi: 10.1016/j.molp.2019.01.007. Anti-streptavidin-HRP and anti-his-HRP antibodies were used to detect purified proteins. Anti-streptavidin-HRP has been validated for detection of BioDiaAlk (doi.org/10.1038/s41589-018-0116-2). Anti-his-HRP has been validated against non-his-tagged AtPCO4 recombinant protein.

Eukaryotic cell lines

Policy information about cell lines and Sex and Gender in Research

Cell line source(s)	Saccharomyces cerevisiae strain BY4742 (Matα; his3-Δ1; leu2-Δ0; lys2-Δ0; ura3-Δ0)
Authentication	Authentication based on auxotrophies
Mycoplasma contamination	We did not test the cells for mycoplasma contamination
Commonly misidentified lines<br>(See ICLAC register)	N/A

Dual use research of concern

Policy information about dual use research of concern

Hazards

Could the accidental, deliberate or reckless misuse of agents or technologies generated in the work, or the application of information presented in the manuscript, pose a threat to:

No	Yes	
☒	☐	Public health
☒	☐	National security
☒	☐	Crops and/or livestock
☒	☐	Ecosystems
☒	☐	Any other significant area

Experiments of concern

Does the work involve any of these experiments of concern:

No	Yes	
☒	☐	Demonstrate how to render a vaccine ineffective
☒	☐	Confer resistance to therapeutically useful antibiotics or antiviral agents
☒	☐	Enhance the virulence of a pathogen or render a nonpathogen virulent
☒	☐	Increase transmissibility of a pathogen
☒	☐	Alter the host range of a pathogen
☒	☐	Enable evasion of diagnostic/detection modalities
☒	☐	Enable the weaponization of a biological agent or toxin
☒	☐	Any other potentially harmful combination of experiments and agents

Plants

Seed stocks	A. thaliana Columbia-0 (Col-0) CS70000 from ABRC; erfVII (rap2.2-1 rap2.3-1 rap2.12-1 hre1 hre2) described in https://doi.org/10.1104/pp.114.244723; prt6-5 is SALK_051088 from NASC; ate1/2 described in https://doi.org/10.1073/pnas.0906404106 pco mutants described in https://doi.org/10.1111/pce.14440. roGFP2-Orp1 described in https://doi.org/10.1111/nph.15550.
Novel plant genotypes	New plant genotypes were generated using Agriobacterium mediated transformation as detailed in M&M.
Authentication	Genotype autentication was performed by PCR
