# Supplementary material for: H2O2 repurposes plant O2 sensing to regulate post-hypoxia responses
Source: Nature. 2026 Apr 22;653(8116):1130–8. doi: 10.1038/s41586-026-10366-1 (PMC13216066; doi:10.1038/s41586-026-10366-1)
Supplement: Supplementary file 3 — Extended legend for Fig. 1 including replicate numbers and box plot. [file 41586_2026_10366_MOESM3_ESM.docx]

**Fig. 1. ERFVIIs mediate plant tolerance upon reoxygenation** **(Extended legend)**

(**a**) Schematic diagram of experimental design; seven-day old Arabidopsis seedlings were exposed to severe hypoxia (0.1 % O_2_) or air (21 % O_2_), in darkness, for 24 h and subsequently returned to aerobic conditions for 4 days. (**b**) Phenotype of Col-0 and *erfVII* seedlings before and after hypoxia treatment or air control, and after 4 days reoxygenation (scale bar, 1 cm). (**c**) Percentage of alive, damaged or dead seedlings after 4 days of post-hypoxia reoxygenation or air control. Two-sided *χ*^2^ test followed by a post-hoc test with a Bonferroni correction was used to analyse this dataset (p < 0.05). Asterisks indicate statistical differences between Col-0 and *erfVII*. (**d**) Growth rate of primary roots after 4 days of reoxygenation or air control (Col-0 in air, *n =*32; Col-0*-*in reoxygenation, *n* = 31; *erfVII* in air, *n =*30; *erfVII*in reoxygenation, *n* = 29). (**e**) Fresh weight per plate after 4 days of reoxygenation or air control (*n*= 5). (**f**) DAB staining of Col-0 and *erfVII* seedlings following exposure to 0.1 % or 21 % O_2_ for 24 h in darkness, and subsequently returned to aerobic conditions for 0, 1 or 6 h (scale bar, 0.5 cm). (**g**) Quantification of DAB staining intensity represented in arbitrary unit (au) (Col-0 in air at 1 h and 6 h, reoxygenation at 6 h and *erfVII* in air at 0 h, 1 h and 6 h, reoxygenation at 1 h and 6 h, *n*= 8; Col-0 in air at 0 h and reoxygenation at 1 h, *n* = 7; Col-0 and *erfVII* in reoxygenation at 0 h, *n* = 5). Two-way ANOVA followed by Tukey’s HSD test (p < 0.05) was applied to analyse the datasets in **d-g**; different letters indicate statistically distinct groups (p < 0.05). (**h-j**) Multi-well fluorimetry of cytosolic oxidative stress using seven-day old Arabidopsis seedlings expressing the biosensor roGFP2-Orp1 in Col-0, *erfVII* and *prt6* background over time, each normalised to the baseline oxidative state before the start of hypoxic treatment (*n* = 28). (**i-j**) Amplitudes of late hypoxic roGFP2-Orp1 oxidation before reoxygenation (purple arrow, **i**) and maximum oxidative burst during reoxygenation (green arrow, **j**), each normalized to the baseline oxidative state before the start of hypoxic treatment (*n* = 28). In **d**, **e**, **g**, boxplots indicate median (middle line), 25^th^ and 75^th^ percentiles (box limits), whiskers denote the 1.5x interquartile range; outliers are shown as individual points.
