# Supplementary material for: H2O2 repurposes plant O2 sensing to regulate post-hypoxia responses
Source: Nature. 2026 Apr 22;653(8116):1130–8. doi: 10.1038/s41586-026-10366-1 (PMC13216066; doi:10.1038/s41586-026-10366-1)
Supplement: Supplementary file 7 — Supplementary Table 7: DNA sequences generated in this study for design and assembly of transgenic constructs. Supplementary Table 8: list of primers used for generation and screening of RAP2.3–nLuc and RAP2.3–GFP transgenic line. Supplementary Table 9: list of primers for qRT-PCR used in this study. Supplementary Table 10: list of primers for ChIP–qPCR used in this study. [file 41586_2026_10366_MOESM7_ESM.docx]

**SUPPLEMENTARY TABLES**

**Supplementary Table 7.** DNA sequences generated in this study for the design and ssembly of transgenic constructs.

| **Construct** | **Sequence** |
| --- | --- |

| **nLuc-intron** | ATGGTTTTCACCCTTGAGGACTTCGTTGGAGATTGGAGACAGACCGCTGGATACAACCTTGATCAGGTGTTGGAGCAAGGTGGGGTGTCATCTTTGTTCCAGAACCTCGGAGTTAGCGTGACCCCTATCCAGAGAATCGTTCTCTCTGGTGAGAACGGGCTCAAGATCGATATCCACGTGATCATCCCTTACGAGGGACTTAGCGGAGATCAGATGGGACAGATCGAGAAGATTTTCAAGGTGGTGTACCCTGTGGACGACCACCACTTCAAGGTTATCCTCCATTACGGtaaatatttagggattgactttagtttgagcgattgaattggttagaaattaagtttcgattctatttagctctgttcatagatacctttatctgcttacgattttgattgttgtataaagcatgaggtttgatttgtgtgatatttatatgcatatgaggttgagtgatgagacttgatttgtgtgatgagatttgattagagattgttgatgagttatttatagaagaactcttttgttttgttgttgtttcactactagGAACCCTCGTGATCGATGGTGTGACCCCAAACATGATCGACTACTTCGGTAGACCGTACGAGGGAATCGCTGTGTTCGATGGAAAGAAGATTACCGTCACTGGGACCCTCTGGAACGGGAACAAGATTATCGATGAGAGGCTCATCAACCCGGACGGCTCACTTTTGTTCAGAGTGACTATCAACGGTGTGACCGGTTGGAGACTTTGCGAGAGAATTTTGGCTGGAAGCAGCGGAGCTATCGCCATGGAATGA |
| --- | --- |
| **GW-nLuc** | CTCCCATATGGTCGACCTGCAGGCGGCCGCACTAGTGATATC**ACAAGTTT**GTACAAAAAAGCTGAACGAGAAACGTAAAATGATATAAATATCAATATATTAAATTAGATTTTGCATAAAAAACAGACTACATAATACTGTAAAACACAACATATCCAGTCACTATGGCGGCCGCATTAGGCACCCCAGGCTTTACACTTTATGCTTCCGGCTCGTATAATGTGTGGATTTTGAGTTAGGATCCGGCGAGATTTTCAGGAGCTAAGGAAGCTAAAATGGAGAAAAAAATCACTGGATATACCACCGTTGATATATCCCAATGGCATCGTAAAGAACATTTTGAGGCATTTCAGTCAGTTGCTCAATGTACCTATAACCAGACCGTTCAGCTGGATATTACGGCCTTTTTAAAGACCGTAAAGAAAAATAAGCACAAGTTTTATCCGGCCTTTATTCACATTCTTGCCCGCCTGATGAATGCTCATCCGGAATTCCGTATGGCAATGAAAGACGGTGAGCTGGTGATATGGGATAGTGTTCACCCTTGTTACACCGTTTTCCATGAGCAAACTGAAACGTTTTCATCGCTCTGGAGTGAATACCACGACGATTTCCGGCAGTTTCTACACATATATTCGCAAGATGTGGCGTGTTACGGTGAAAACCTGGCCTATTTCCCTAAAGGGTTTATTGAGAATATGTTTTTCGTCTCAGCCAATCCCTGGGTGAGTTTCACCAGTTTTGATTTAAACGTGGCCAATATGGACAACTTCTTCGCCCCCGTTTTCACCATGGGCAAATATTATACGCAAGGCGACAAGGTGCTGATGCCGCTGGCGATTCAGGTTCATCATGCCGTCTGTGATGGCTTCCATGTCGGCAGAATGCTTAATGAATTACAACAGTACTGCGATGAGTGGCAGGGCGGGGCGTAAACGCGTGGATCCGGCTTACTAAAAGCCAGATAACAGTATGCGTATTTGCGCGCTGATTTTTGCGGTATAAGAATATATACTGATATGTATACCCGAAGTATGTCAAAAAGAGGTGTGCTATGAAGCAGCGTATTACAGTGACAGTTGACAGCGACAGCTATCAGTTGCTCAAGGCATATATGATGTCAATATCTCCGGTCTGGTAAGCACAACCATGCAGAATGAAGCCCGTCGTCTGCGTGCCGAACGCTGGAAAGCGGAAAATCAGGAAGGGATGGCTGAGGTCGCCCGGTTTATTGAAATGAACGGCTCTTTTGCTGACGAGAACAGGGACTGGTGAAATGCAGTTTAAGGTTTACACCTATAAAAGAGAGAGCCGTTATCGTCTGTTTGTGGATGTACAGAGTGATATTATTGACACGCCCGGGCGACGGATGGTGATCCCCCTGGCCAGTGCACGTCTGCTGTCAGATAAAGTCTCCCGTGAACTTTACCCGGTGGTGCATATCGGGGATGAAAGCTGGCGCATGATGACCACCGATATGGCCAGTGTGCCGGTCTCCGTTATCGGGGAAGAAGTGGCTGATCTCAGCCACCGCGAAAATGACATCAAAAACGCCATTAACCTGATGTTCTGGGGAATATAAATGTCAGGCTCCCTTATACACAGCCAGTCTGCAGGTCGACCATAGTGACTGGATATGTTGTGTTTTACAGTATTATGTAGTCTGTTTTTTATGCAAAATCTAATTTAATATATTGATATTTATATCATTTTACGTTTCTCGTTCAGCTTTCTTGTACAAAGTGGTGATAAAAAAATGGTTTTCACCCTTGAGGACTTCGTTGGAGATTGGAGACAGACCGCTGGATACAACCTTGATCAGGTGTTGGAGCAAGGTGGGGTGTCATCTTTGTTCCAGAACCTCGGAGTTAGCGTGACCCCTATCCAGAGAATCGTTCTCTCTGGTGAGAACGGGCTCAAGATCGATATCCACGTGATCATCCCTTACGAGGGACTTAGCGGAGATCAGATGGGACAGATCGAGAAGATTTTCAAGGTGGTGTACCCTGTGGACGACCACCACTTCAAGGTTATCCTCCATTACGGAACCCTCGTGATCGATGGTGTGACCCCAAACATGATCGACTACTTCGGTAGACCGTACGAGGGAATCGCTGTGTTCGATGGAAAGAAGATTACCGTCACTGGGACCCTCTGGAACGGGAACAAGATTATCGATGAGAGGCTCATCAACCCGGACGGCTCACTTTTGTTCAGAGTGACTATCAACGGTGTGACCGGTTGGAGACTTTGCGAGAGAATTTTGGCTGGAAGCAGCGGAGCTATCGCCATGGAATGACCGCGGCCATGCTAGAGTCCGCAAAAATCACCAGTCTCTCTCTACAAATCTATCTCTCTCTATTTTTCTCCAGAATAATGTGTGAGTAGTTCCCAGATAAGGGAATTAGGGTTCTTATAGGGTTTCGCTCATGTGTTGAGCATATAAGAAACCCTTAGTATGTATTTGTATTTGTAAAATACTTCTATCAATAAAATTTCTAATTCCTAAAACCAAAATCCAGTGACCTGCAGGCATGCGACGTCGGGCCCA |
| **RAP2.12 with 3xFLAG tag and 3xHA tag** | ATGTGTGGAGGAGCTATAATATCCGATTTCATTCCACCGCCGAGGTCTCGCCGTGTTACTAGCGAGTTTATTTGGCCGGATCTGAAGAAGAATTTGAAAGGATCGAAGAAAAGCTCGAAGAATCGTTCGGATTATAAGGACCATGATGGGGACTATAAGGATCACGATATTGACTACAAAGATGACGACGACAAATTCGACGTCGGTGATGTTTTCGCCGATGTGAAACCATTCGTTTTCACTTCGACTCCAAAACCCGCCGTCTCCGCCGCTGCGGAAGGTTCAGTTTTTGGTAAGAAAGTTACTGGCTTGGATGGGGACGCTGAGAAATCTGCAAATAGGAAGAGGAAGAATCAGTACCGAGGGATTAGGCAACGTCCTTGGGGAAAATGGGCTGCTGAGATACGTGATCCAAGGGAAGGTGCTAGAATCTGGCTTGGAACGTTCAAGACAGCTGAGGAAGCTGCTAGAGCTTACGATGCTGCAGCGCGGAGAATCCGTGGATCTAAAGCTAAGGTGAATTTCCCTGAAGAAAACATGAAGGCTAATTCTCAGAAACGCTCTGTGAAGGCTAATCTTCAGAAACCAGTGGCTAAACCTAACCCTAACCCAAGTCCAGCTTTGGTTCAGAACTCGAACATCTCCTTTGAAAATATGTGTTTCATGGAGGAGAAACACCAAGTGAGCAACAACAACAACAACCAGTTTGGGATGACAAACTCCGTTGATGCTGGATGTAATGGGTATCAGTATTTCAGCTCTGACCAGGGTAGTAATTCTTTCGATTGTTCGGAGTTTGGTTGGAGCGATCAAGCTCCGATAACTCCCGACATCTCTTCTGCGGTTATCAACAACAACAACTCAGCTCTGTTCTTTGAGGAAGCCAATCCAGCTAAGAAGCTCAAGTCTATGGATTTCGAGACACCTTACAACAACACTGAATGGGACGCTTCACTGGATTTCCTCAACGAAGATGCTGTAACGACTCAGGACAATGGTGCAAACCCTATGGACCTATGGAGTATTGATGAAATTCATTCCATGATTGGAGGAGTCTTCGGTGGAGGCGGTTCAGCATACCCTTACGATGTTCCTGACTATGCGGGCTATCCCTATGACGTCCCGGACTATGCCGGCTACCCTTACGACGTTCCAGATTACGCT |

**Supplementary Table 8.** List of primers for generation and screening of RAP2.3-nLuc and RAP2.3-GFP transgenic line.

| **Primer Name** | **Primer** |
| --- | --- |
| RAP2.3∆stop Fw | AAAAAAGCAGGCTCCATGTGTGGCGGTGCTATTAT |
| RAP2.3∆stop Rv | CAAGAAAGCTGGGTGCTCATACGACGCAATGACAT |
| AttB1 | GGGGACAAGTTTGTACAAAAAAGCAGGCTCC |
| AttB2 | CACCCAGCTTTCTTGTACAAAGTGGTCCCC |
| nLuc Rv | AGTCCCTCGTAAGGGATGAT |
| GFP Rv | ACAACTCCAGTGAAAAGTTC |

**Supplementary Table 9.** List of primers for qRT-PCR used in this study.

| **Gene** | **AGI Code** | **Forward Primer** | **Reverse Primer** |
| --- | --- | --- | --- |
| *UBQ10* | *AT4G05320* | GGCCTTGTATAATCCCTGATGAATAAG | AAAGAGATAACAGGAACGGAAACATAGT |
| *LBD41* | *AT3G02550* | TGAAGCGCAAGCTAACGCA | ATCCCAGGACGAAGGTGATTG |
| *ADH1* | *AT1G77120* | TATTCGATGCAAAGCTGCTGTG | CGAACTTCGTGTTTCTGCGGT |
| *PDC1* | *AT4G33070* | CACAGAATCTTCAATGTTCTTACC | CCATGATAAAGCGTACATGGAA |
| *SUS1* | *AT5G20830* | ACGCTGAACGTATGATAACGCG | AACCCTGGAAAGCAAGGCAAG |
| *HRA1* | *AT3G10040* | ACAACCACCGCAACAGAATCC | TCTCCGCAATTCTCGCCAT |
| *HB1* | *AT2G16060* | AATGATTTATAACTGCAGGTGGC | TCATAAGCCTGACCCCAAGC |
| *SAD6* | *AT1G43800* | TTGGCAACCCGCTTCTTTCTTACC | TTTCCCTCAGCTCACGAACCTG |
| *SUS4* | *AT3G43190* | AACGCAGAACGTGTAATAACG | CTCGGAGTGATGTTGAGTCC |
| *PCO1* | *AT5G15120* | ATTGGGTGGTTGATGCTCCAATG | ATGCATGTTCCCGCCATCTTC |
| *PCO2* | *AT5G39890* | CTTCGAGCCGTTTTGGATGA | ACGTCACTAACGGAGATCGTCC |
| *HUP7* | *AT1G43800* | TTGGCAACCCGCTTCTTTCTTACC | TTTCCCTCAGCTCACGAACCTG |
| *HUP9* | *AT5G10040* | TCATCGGCGGACATAGCAA | ATCATCAACCACCCAACCTCC |
| *CRK36* | *AT4G04490* | CCGGATGCGGAGGAGGATTT | TACCGCTATCTCTTGCCCGC |
| *GSTU24* | *AT1G17170* | GAGACTTGGCCCGACAATAA | CTCGCCGTAACATTCACCTT |
| *ZAT12* | *AT5G59820* | CATCACAACTACTATCACACCAAACTC | ATCCACCGTCGACTTGATCT |
| *nLuc* | *NA* | CCAGAACCTCGGAGTTAGCG | CTGTCCCATCTGATCTCCGC |

**Supplementary Table 10.** List of primers for ChIP-qPCR used in this study.

| **Gene** | **AGI Code** | **Forward Primer** | **Reverse Primer** |
| --- | --- | --- | --- |
| *UBQ10* | *AT4G05320* | TCCCTCCCTTTAAGCACCAG | TCCGGTCCTAGATCATCAGTTCA |
| *EIF4AI* | *AT3G13920* | TGTTTTGCTTCGTTTCAAGGA | GCATTTTCCCGATTACAAC |
| *ADH1* | *AT1G77120* | GCAAAACCAAATACGCCCC | TAATCTGTCCGGTGGTAGAC |
| *LBD41* | *AT3G02550* | GAGAGAGTCACAAAGATCCGCCC | GAAGAACTGGGGCCCACACTTAG |
| *HB1* | *AT2G16060* | CCATGTGCTCTGTACTGGTAATGGA | TTATACCACTTGGTGTGGTTGGC |
| *HRA1* | *AT3G10040* | GCAGTGGTTTTGGGAGCCGT | TTTGCCAAAACCAGCCCCTTG |
| *ZAT12* | *AT5G59820* | TACGCGGTGTCGCAAATCGT | TGGGTAAGGAAGTGGCAGCG |
| *ATH8* | *AT1G69880* | AGGTTTACATGCAACTTTCCGCT | CTGGTGGACCAAGTAGCCGT |
| *GSTU24* | *AT1G17170* | TCAAGTGCGCCAAAAGGAAAGA | AGGGGTTTTGAATCGCATTTTGCT |
